# Genome-Wide Identification and Expression of NF-YC Transcription Factors in Blueberry Under Abiotic Stress Conditions

**DOI:** 10.3390/ijms26178507

**Published:** 2025-09-01

**Authors:** Xiang Zhang, Jiajie Yu, Xiuyue Xu, Baofeng Zhang, Jiahuan Huang, Bo Liu

**Affiliations:** 1 School of Agriculture, Liaodong University, Dandong 118003, China; zhangxiang@liaodongu.edu.cn (X.Z.); yujiajie@liaodongu.edu.cn (J.Y.); 18845127662@163.com (B.Z.); huangjiahuan2012@126.com (J.H.); 2 Forestry College, Northeast Forestry University, Harbin 150040, China; 15663593162@163.com

**Keywords:** NF-YC, blueberry (*Vaccinium corymbosum*), bioinformatic analysis, stress response

## Abstract

Nuclear Factor Y C (NF-YC) transcription factors (TFs) are central regulators of plant development and stress adaptation. However, there remains a gap in identifying NF-YC gene family members in blueberry (*Vaccinium corymbosum*), a globally significant fruit crop renowned for its nutritional value and good adaptability. In this study, a total of 31 *NF-YC* genes (designated *VcNF-YC1–31*) were identified in the blueberry genome, and their basic physicochemical properties, gene structures, motif patterns, and conserved domains were investigated using bioinformatic methods. The *cis*-acting elements in the promoters of *VcNF-YC*s were mainly enriched in phytohormone signaling, metabolism, and stress response. qRT-PCR analysis showed that *VcNF-YC*s were expressed at higher levels in leaves than in roots and stems. Transcriptional profiling revealed rapid upregulation of 24, 25, and 16 *VcNF-YC* genes upon ABA, salt, and cold treatments, respectively, indicating stress-specific induction patterns. The results of the yeast transformation assay revealed that VcNF-YC10 and VcNF-YC15 lacked transcription-activating activity. The results of tobacco leaf injection revealed that these two TFs were localized in the nucleus. These findings indicate the potentially important roles in abiotic stress responses of blueberry, offering potential targets for molecular breeding to enhance plant resilience.

## 1. Introduction

Transcription factors (TFs) are DNA-binding proteins that serve as central regulators of gene expression [1]. As sessile organisms exposed to fluctuating environments, plants have evolved sophisticated mechanisms to withstand diverse abiotic stresses, including drought, waterlogging, low temperature, high temperature, salt stress, heavy metal stress, etc. [2,3,4]. Therefore, studying TFs in plant science is critically important for understanding stress response mechanisms.

Nuclear Factor Y (NF-Y) TFs, also called CBF (CCAAT-binding factor) or HAP (hem activator protein), exist widely in plants, yeast, mammals, and other eukaryotes [5]. They function by binding to CCAAT boxes in the promoters of downstream genes [6]. The TF members in this family consist of three subunits: NF-YA (CBF-B/HAP2), NF-YB (CBF-A/HAP3), and NF-YC (CBF-C/HAP5) [7]. NF-Y TFs usually function by forming heterodimers or heterotrimers. Although named systematically, these three subunits share limited structural homology, despite functioning as a complex. The structural domain of NF-YA protein possesses two helices (A1 and A2) with independent functions (The A1 domain mediates subunit interactions, while the A2 domain binds to the CCAAT box.) [8]. Overexpression of *BnNF-YA3* in rapeseed delayed seed germination and root elongation under ABA and salinity stress, which indicated ABA-dependent stress regulation [9]. NF-YB includes a histone-fold domain (HFD) for dimerization with NF-YC and a conserved B-domain for trimer stabilization. In maize, overexpression of *ZmNF-YB2* conferred drought tolerance by enhancing photosynthesis and root architecture [10]. NF-YC proteins are characterized by a histone-fold motif (HFM) resembling histone H2A, which facilitates dimerization with the NF-YB subunit (histone H2B-like) to form a stable NF-YB/NF-YC heterodimer—a prerequisite for recruiting NF-YA (histone H3-like) to assemble the functional trimer [11]. This trimeric complex binds to the CCAAT *cis*-elements in downstream gene promoters, which is an important mechanism conserved across eukaryotes.

*NF-Y* genes were identified for the first time in the 1990s in the model plant *Arabidopsis* [12]. The successful cloning and characterization of *NF-Y* genes in other species, such as tomato and tobacco, gave early evidence of their conservation and potentially important functional roles. Over the decades, numerous studies have proven the multiple biological functions of NF-Y TFs in various biological processes, including gamete development, embryogenesis, seed germination, root morphogenesis, flowering, fruiting, hypocotyl elongation, photosynthesis, starch biosynthesis, and abscisic acid (ABA) signaling [13,14,15,16,17,18,19,20,21,22,23,24]. Furthermore, numerous studies have shown the involvement of NF-YC TFs in the abiotic stress responses of plants. In soybean (*Glycine max*), overexpression of *GmNF-YC14* improved the drought tolerance of the transgenic plants by upregulating ABA biosynthesis-related genes and stomatal closure regulators, while the CRISPR-Cas9 mutants exhibited hypersensitivity to water deficit [25]. In ginseng (*Panax ginseng*), *PgNF-YC07-04* was significantly upregulated upon salt stress, which indicated the important role of this gene in salt stress response [26]. In grapevine (*Vitis vinifera* and *Vitis amurensis*), the expression of *VaNF-YA6*, *VaNF-YB5*, *VvNF-YA3*, *VvNF-YA5*, and *VvNF-YC2* genes was significantly induced by salt and drought treatments. Additionally, overexpression of *VaNF-YA6* in grapevine and *Arabidopsis* significantly increased tolerance of the transgenic plants to salt and drought stresses by increasing the expression of *VvSOS2*, *VvSOS3*, *VvABF3*, and *VvCPK6* genes, as well as antioxidant enzyme activities and the contents of other protective substances [27].

With the development of bioinformatics, *NF-YC* genes have been identified in numerous plant species from a genome-wide perspective. A total of 14, 16, 15, 9, 6, 9, 8, and 8 *NF-YC* genes were identified in wheat, tobacco, soybean, potato, peach, jujube, melon, and grape, respectively [28,29,30,31,32,33,34,35]. However, *NF-YC* genes remain to be identified in blueberry. In woody perennials, the expansion of the NF-YC family may facilitate the formation of distinct protein complexes to fine-tune developmental processes such as flowering, which is crucial for perennial growth cycles. For instance, in apple (*Malus domestica*), a comprehensive genome-wide analysis identified 14 *MdNF-YC* genes [36]. This number was slightly higher than that found in *Arabidopsis* (13 *AtNF-YC*s) but similar to the expansion observed in other woody species. The expansion in apple has been linked to functional diversification, with specific MdNF-YC members (e.g., MdNF-YC3, MdNF-YC7, and MdNF-YC14) demonstrated to interact with MdNF-YB18 to regulate flowering time via the activation of *MdFT* expression. Blueberry plant (*Vaccinium* spp.), a perennial shrub native to North America, is renowned for its nutrient-rich berries, which are rich in antioxidants, vitamins, and dietary fiber. Globally, blueberry planting and production are now facing serious challenges from both regular and unpredictable abiotic stress events. For instance, in 2022, blueberry production experienced a decrease due to drought and generally unfavorable weather conditions although planting area was stable [37]. Genome-wide identification and characterization of the NF-YA gene family in blueberry was recently reported, providing valuable insights into the NF-Y subunit [38]. However, a comprehensive analysis of the NF-YC subunit family in the blueberry genome is still lacking. Given the important roles of *NF-YC* genes in abiotic stress response, it is of great value and necessity to identify the *NF-YC* genes in blueberry. Therefore, the purpose of this study was to conduct a genome-wide identification and characterization of the NF-YC transcription factor family in blueberry. In this study, we identified NF-YC family members in the blueberry genome. Their protein sequences, basic physicochemical properties, chromosome location, gene structures, and evolutionary relationships were analyzed using bioinformatic tools. Their tissue-specific expression patterns and expression profiles under salt, ABA, and cold treatments were investigated using qRT-PCR assays. This study provides a foundational resource for understanding the role of *VcNF-YC* genes in blueberry stress biology and offers potential genetic targets for the breeding of stress-resilient blueberry cultivars.

## 2. Results

### 2.1. Identification and Physicochemical Properties of VcNF-YC TFs

To identify the NF-YC members in the blueberry genome, a BlastP search was performed in the *V. corymbosum* cv. Duke v1 genome database using 14 AtNF-YC protein sequences as queries. After Pfam analysis and HMMER-based predictions, a total of 31 *VcNF-YC* genes were identified in the blueberry genome, which were designated *VcNF-YC1*–*C31* according to the order of their location on scaffolds. Their physicochemical properties revealed substantial diversity (Table 1). The VcNF-YC proteins consisted of 65 (VcNF-YC8) to 367 (VcNF-YC30) amino acids; therefore, the molecular weight of these proteins also exhibited differences, ranging from 7557.92 (VcNF-YC8) to 41,228.12 (VcNF-YC30) Da. The isoelectric points of the VcNF-YCs ranged from 3.97 (VcNF-YC20) to 9.16 (VcNF-YC8), indicating both acidic and basic isoforms. The instability indexes ranged from 42.72 (VcNF-YC17) to 74.46 (VcNF-YC4), indicating that all the VcNF-YCs were unstable proteins. Aliphatic indices (AI) ranged from 59.32 (VcNF-YC31) to 108 (VcNF-YC8), indicating divergent thermostability profiles. The majority (25/31) of VcNF-YCs exhibited moderate AI values (60–85), suggesting stable folding under physiological temperatures. The grand average of hydropathy (GRAVY) values of VcNF-YCs were universally negative, confirming hydrophilic characteristics across the family.

### 2.2. Multiple Sequence Alignment and Phylogenetic Relationships of VcNF-YCs

**Figure 1:**
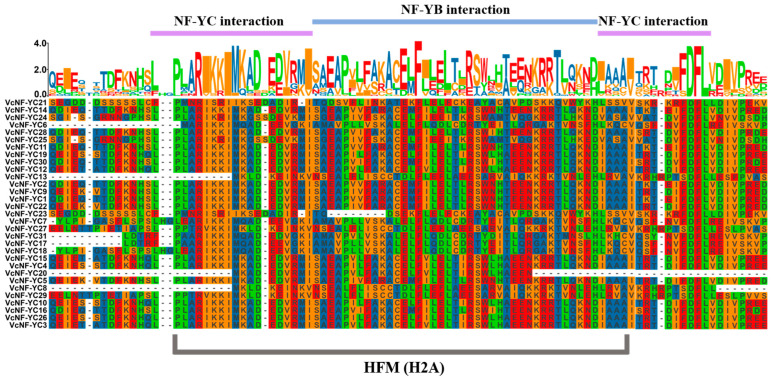
Multiple sequence alignment of VcNF-YCs. Amplified colored letters above protein sequences indicate a motif. Different colored letters represent different amino acids.

**Figure 2:**
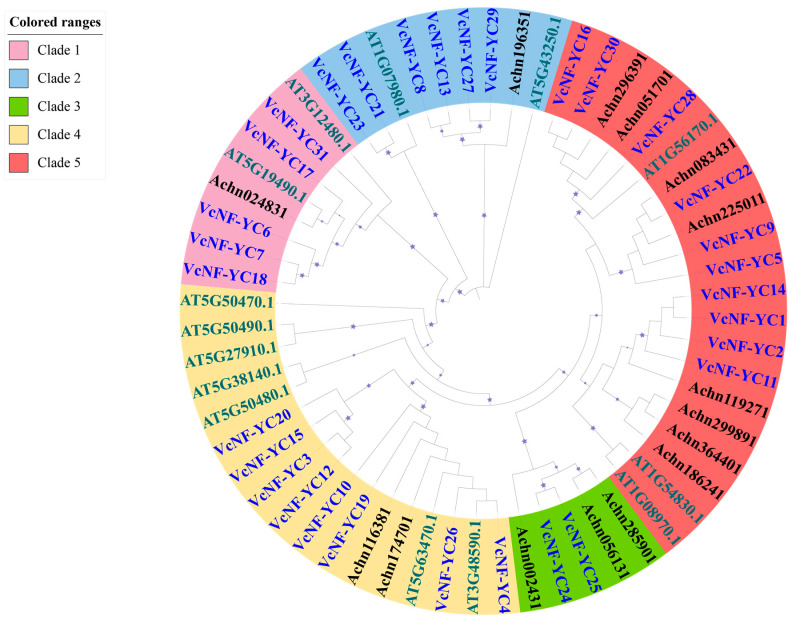
Phylogenetic analysis of NF-YC members in blueberry (*Vaccinium corymbosum*), *Arabidopsis* (*Arabidopsis thaliana*), and Chinese kiwi fruit (*Actinidia chinensis*). The phylogenetic tree (1000 bootstrap replicates) was constructed using TBtools. VcNF-YCs are shown in blue, AtNF-YCs are shown in green, and AcNF-YCs are shown in black.

Results of the multiple sequence alignment analysis are displayed in Figure 1. All VcNF-YCs possess two NF-YC interaction domains (at the N-terminal and the C-terminal) and one NF-YB interaction domain. Furthermore, the phylogenetic relationships of VcNF-YCs were investigated by constructing a phylogenetic tree via TBtools (v2.136) software based on their full-length protein sequences (Figure 2). To improve the reliability of the results, the NF-YC members in *Arabidopsis* and Chinese kiwifruit were also introduced into the phylogenetic tree. The results showed that VcNF-YCs were classified into five clades: Clade 1 (VcNF-YC18, -7, -6, -17, -31), Clade 2 (VcNF-YC23, -21, -8, -13, -27, -29), Clade 3 (VcNF-YC25, -24), Clade 4 (VcNF-YC4, -26, -19, -10, -12, -3, -15, -20) and Clade 5 (VcNF-YC16, -30, -28, -22, -9, -5, -14, -1, -2, -11). The clades were defined based on evolutionary relatedness in the phylogenetic tree.

### 2.3. Gene Structures and Conserved Motifs of VcNF-YCs

**Figure 3:**
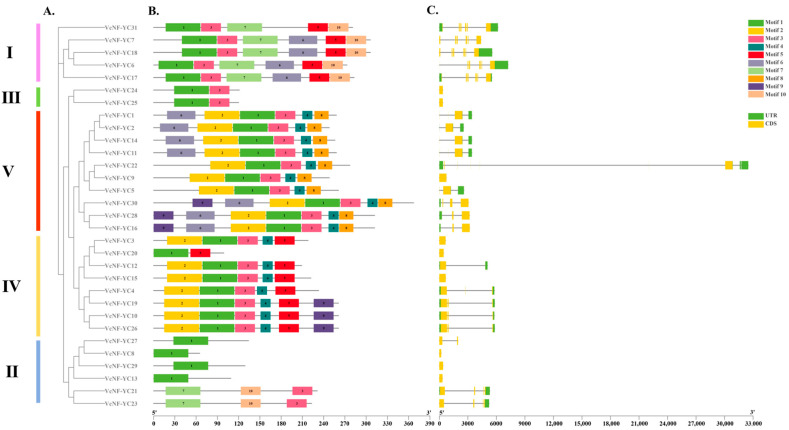
Conserved domains and gene structures of VcNF-YC members aligned by their phylogenetic relationships. (**A**) Phylogenetic tree constructed based on the full-length sequences of the VcNF-YCs. (**B**) Motif composition of VcNF-YCs. Different colored boxes symbolize different motifs. (**C**) Exon–intron structures of the *VcNF-YC*s. Yellow boxes indicate exons, green boxes indicate untranslated regions (UTRs), and black lines indicate introns.

The results of the gene structure and conserved motif analyses are displayed with VcNF-YCs arranged based on their phylogenetic relationships (Figure 3A). Generally, the VcNF-YCs in the same clade share similar gene structures (Figure 3C) and conserved motif patterns (Figure 3B); for instance, the VcNF-YCs in Clade I all have Motifs 1, 3, 5, 7, and 10. In Clade II, VcNF-YC27, -8, -29, and -13 have only Motif 1, while VcNF-YC21 and VcNF-YC23, which had closer phylogenetic relationships in this clade, have Motifs 7, 10, and 3. In Clade III, VcNF-YC24 and VcNF-YC25 have Motifs 1 and 3. In Clade IV, all members have Motifs 2, 1, 3, 4, and 5, except VcNF-YC20, while VcNF-YC19, -10, and -26 have Motif 9. The VcNF-YCs in Clade V have Motifs 2, 1, 3, 4, and 8. As for exon–intron patterns, the *VcNF-YC*s in Clade I have multiple exons and introns. In Clade II, *VcNF-YC8*, *-29*, and *-13* have only one exon and no introns, indicating different exon–intron patterns compared to the other members of the clade. In Clade III, *VcNF-YC24* and *VcNF-YC25* have only one exon and no introns. The sequences of these motifs are listed in Table S1.

### 2.4. Intraspecies Collinearity of VcNF-YCs

**Figure 4:**
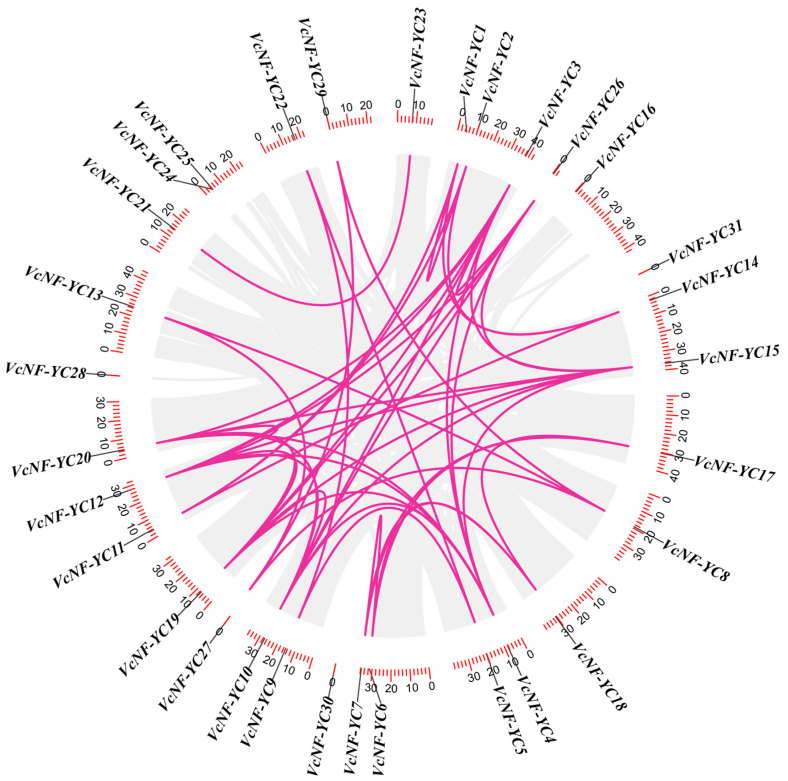
Intraspecies collinear relationships between *VcNF-YC* genes. Pink lines indicate collinear relationships, and grey lines indicate all of the collinear backgrounds in the blueberry genome.

Intraspecies collinearity analysis was conducted to investigate the duplication events in the evolution of *VcNF-YC*s. As shown in Figure 4, a total of 48 collinear *VcNF-YC* gene pairs were identified. *VcNF-YC3*, *-19*, and *-12* occurred with the highest frequency (seven times) in these collinear gene pairs. On the contrary, some *VcNF-YC* genes occurred only once, such as *VcNF-YC21*, *-22*, *-23*, and *-5*. A small proportion (6/31) of *VcNF-YC* genes exhibited no collinear relationships with other family members, including *VcNF-YC24*, *-25*, *-16*, *-31*, *-30*, and *-28*. To explore the evolutionary constraints on the *VcNF-YC*s, the Ka/Ks ratios of the *VcNF-YC* gene pairs were calculated. The results in Table S2 indicate that most (39/48) of the gene pairs underwent purification selection (Ka/Ks < 1.0 or both Ka and Ks were 0). A small proportion (9/48) of the gene pairs underwent positive selection (Ka/Ks > 1.0). Information on the specific gene pairs and Ka/Ks ratios is provided in Table S2.

### 2.5. Interspecies Collinearity of VcNF-YCs

**Figure 5:**
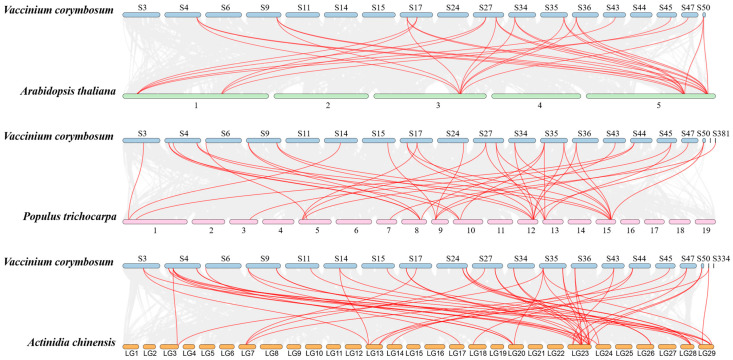
Interspecies collinear relationships between *NF-YC* genes from blueberry and *Arabidopsis*, blueberry and poplar, and blueberry and Chinese kiwi fruit. Red lines indicate collinear relationships, and grey lines indicate all the collinear backgrounds in the blueberry genome. The numbers adjacent to the chromosomes represent chromosome identifiers.

Interspecies collinearity analysis was performed to elucidate the evolutionary trajectory of NF-YC genes across diverse plant lineages. As shown in Figure 5, a total of 31, 40, and 50 collinear *NF-YC* gene pairs were identified between blueberry and *Arabidopsis*, blueberry and poplar, and blueberry and Chinese kiwifruit, respectively. Among *VcNF-YC*s, *VcNF-YC15*, *-4*, *-10*, *-11*, *-12*, and *-20* occurred with the highest frequency (seven times) in the collinear gene pairs. Most (26/31) of the *VcNF-YC* genes exhibited collinearity with their orthologs in the other three species. The details of the collinear gene pairs between *VcNF-YC*s and *AtNF-YC*s, *VcNF-YC*s and *PtNF-YC*s, and *VcNF-YC*s and *AcNF-YC*s are provided in Tables S3–S5, respectively.

### 2.6. Cis-Acting Elements in the Promoters of VcNF-YCs

**Figure 6:**
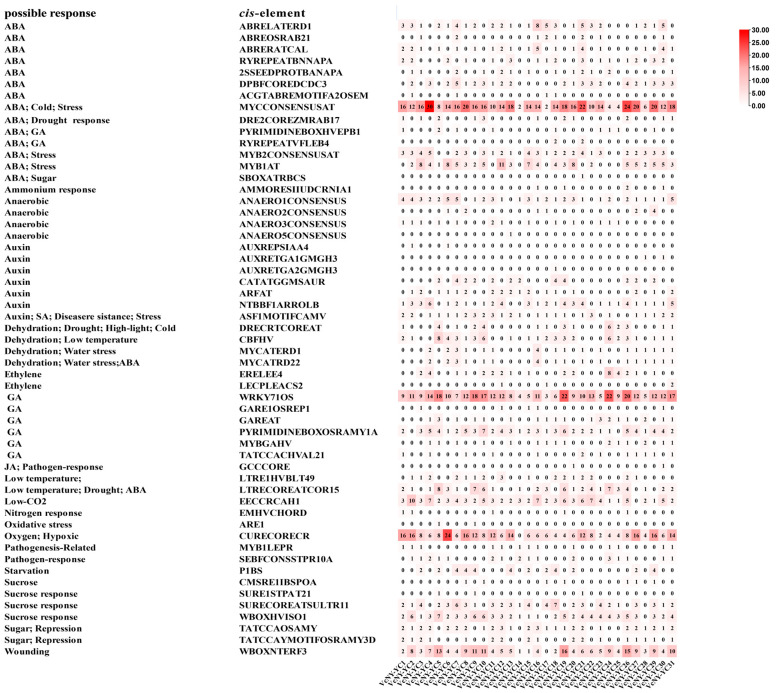
*cis*-acting elements in the promoters of *VcNF-YC* genes. Different colored boxes symbolize different *cis*-acting elements. Scale bar shown to the right measures *cis*-acting element numbers.

In total, 468 types of *cis*-acting elements were identified in the promoters of *VcNF-YC*s using the online tool New PLACE (Figure 6). These *cis*-acting elements were associated with phytohormone signaling (ABA, auxin, ethylene, GA (gibberellin), and JA (jasmonic acid)), metabolism (ammonium response, anaerobic, sucrose, sugar), and stress response (dehydration, low temperature, oxidative stress, pathogen response, wounding). Detailed information, including site name, location, and sequence, is provided in Table S6.

### 2.7. Tissue-Specific Expression Patterns of VcNF-YCs

**Figure 7:**
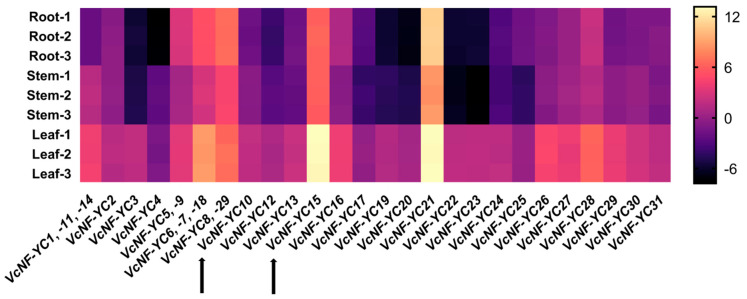
Tissue-specific expression patterns of *VcNF-YC*s. Tissue-specific expression analysis was performed using qRT-PCR of RNA extracted from blueberry roots, stems, and leaves as templates. The relative expression levels of *VcNF-YC*s were calculated using the 2^−ΔΔCt^ method and are presented as log_2_-transformed values. The scale bar is positioned to the right of the heatmap.

qRT-PCR detection was conducted to investigate the expression patterns of *VcNF-YC*s in the roots, stems, and leaves of blueberry plants. As shown in Figure 7, all the genes showed peak expression in leaves, though expression levels varied substantially. For instance, the relative expression level of *VcNF-YC15* in leaves was 108.79 times that in roots and 128.30 times that in stems; the relative expression level of *VcNF-YC21* in leaves was 4.19 times that in roots and 24.32 times that in stems; the relative expression level of *VcNF-YC10* in leaves was 12.59 times that in roots and 5.59 times that in stems; and the relative expression level of *VcNF-YC2* in leaves was 3.49 times that in roots and 3.44 times that in stems. The detailed qRT-PCR data are provided in Table S7.

### 2.8. Expression Patterns of VcNF-YCs Under ABA Treatment

**Figure 8:**
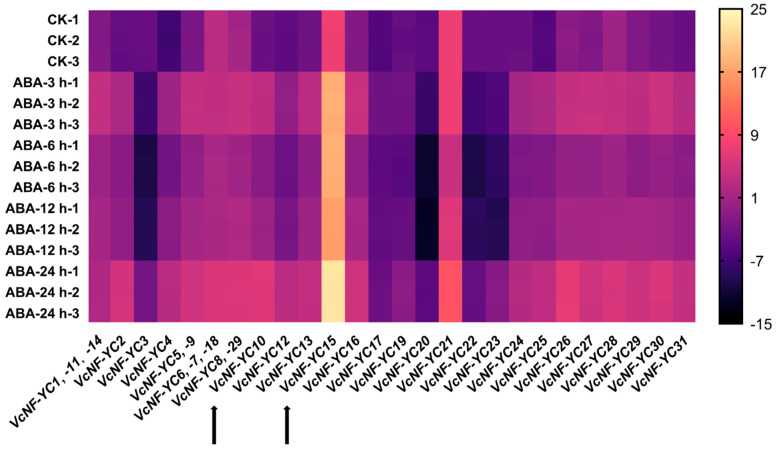
Expression responses of *VcNF-YC*s upon ABA treatment (100 μM ABA solution). The treatment times were 0 h (no ABA treatment, CK), 3 h, 6 h, 12 h, and 24 h. The analysis involved qRT-PCR of RNA extracted from blueberry leaves. The relative expression levels of *VcNF-YC*s were calculated using the 2^−ΔΔCt^ method and are presented as log_2_-transformed values. The scale bar is positioned to the right of the heatmap.

Given that ABA plays a pivotal and comprehensive role in the stress signaling of plants, qRT-PCR analysis was conducted using blueberry leaves as a template to investigate the expression responses of *VcNF-YC*s upon ABA treatment (Figure 8). The results showed that most of the *VcNF-YC*s exhibited significant ABA responsiveness across treatment durations (0 h (CK), 3 h, 6 h, 12 h, and 24 h), with differential induction levels. Overall, initial ABA response triggered upregulation in most (24/31) of the *VcNF-YC* genes, followed by gene-specific expression kinetics over time. For instance, the expression levels of *VcNF-YC2* reached 68.55 times at 3 h, 8.86 times at 6 h, 14.07 times at 12 h, and 670.15 times at 24 h relative to CK. Similarly, *VcNF-YC4* showed transient induction at 3 h, followed by downregulation at 6 h, culminating in a secondary peak at 24 h compared to CK. Some *VcNF-YC* genes were downregulated right after ABA treatment. For instance, the expression levels of *VcNF-YC20* reached 0.15 times at 3 h, 0.01 times at 6 h, the lowest at 12 h, and bounced back to 0.99 times at 24 h relative to CK. All the data for the relative expression levels (log_2_-value) of *VcNF-YC*s under ABA treatment are provided in Table S8.

### 2.9. Expression Patterns of VcNF-YCs Under Salt Treatment

**Figure 9:**
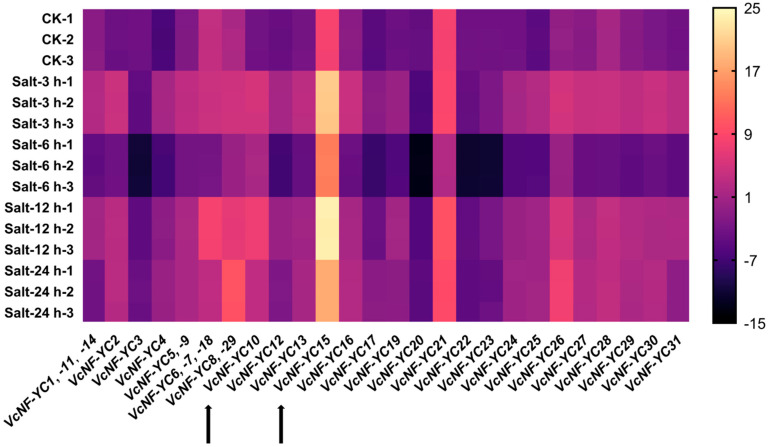
Expression responses of *VcNF-YC*s upon salt treatment. Salt stress was induced using 200 mM NaCl. Treatment times were 0 h (no salt treatment, CK), 3 h, 6 h, 12 h, and 24 h. The analysis involved qRT-PCR of RNA extracted from blueberry leaves. The relative expression levels of *VcNF-YC*s were calculated using the 2^−ΔΔCt^ method and are presented as log_2_-transformed values. The scale bar is positioned to the right of the heatmap.

Blueberry plants were subjected to salt stress treatments (200 mM NaCl solution) across multiple time intervals (0 h (CK), 3 h, 6 h, 12 h, and 24 h), and qRT-PCR detection was performed using blueberry leaves as templates (Figure 9). The results revealed rapid and divergent responses of *VcNF-YC*s upon salt stress. Overall, initial salt response triggered upregulation in most (25/31) of the *VcNF-YC*s, followed by gene-specific expression kinetics over time. For instance, the expression levels of *VcNF-YC2* reached 256.03 times at 3 h, 1.17 times at 6 h, and remained stable at 12 h (93.14 times) and 24 h (89.60 times) relative to CK. The expression levels of *VcNF-YC12* reached 39.95 times at 3 h, 0.14 times at 6 h, 18.60 times at 12 h, and 3.96 times at 24 h relative to CK. A subset of *VcNF-YC*s exhibited immediate downregulation following salt treatment; for instance, the expression levels of *VcNF-YC3* reached 0.43 times at 3 h, 0.006 times at 6 h, 0.40 times at 12 h, and 0.81 times at 24 h relative to CK. All the data for the relative expression levels (log_2_-transformed values) of *VcNF-YC*s under salt treatment are provided in Table S9.

### 2.10. Expression Patterns of VcNF-YCs Under Cold Treatment

**Figure 10:**
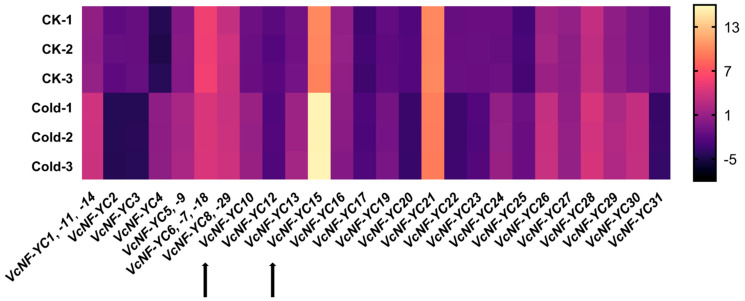
Expression responses of *VcNF-YC*s in response to cold treatment. Treatment condition: 4 °C, 3 h. The analysis involved qRT-PCR of RNA extracted from blueberry leaves. The relative expression levels of *VcNF-YC*s were calculated using the 2^−ΔΔCt^ method and are presented as log_2_-transformed values. The scale bar is positioned to the right of the heatmap.

Given the existence of low-temperature-responsive *cis*-acting elements in the promoters of *VcNF-YC*s, blueberry plants were subjected to cold treatment (4 °C), and qRT-PCR detection was conducted using blueberry leaves as a template to investigate the responses of *VcNF-YC*s to cold stress (Figure 10). Overall, cold stress triggered pronounced expression changes in most (24/31) of the *VcNF-YC*s, with response kinetics varying across the family. Half (16/31) of the *VcNF-YC*s exhibited upregulation in response to cold treatment; for instance, the relative expression level of *VcNF-YC4* at 3 h was 31.64 times relative to CK, while the relative expression level of *VcNF-YC15* at 3 h was 49.66 times relative to CK. A subset of *VcNF-YC* genes was downregulated in response to cold treatment; for instance, the relative expression level of *VcNF-YC3* at 3 h was 0.14 times relative to CK, while the relative expression level of *VcNF-YC22* at 3 h was 0.26 times relative to CK. All the data for the relative expression levels (log_2_-transformed values) of *VcNF-YC*s under cold treatment are provided in Table S10.

### 2.11. VcNF-YC10 and VcNF-YC15 Lack Transcription-Activating Activity in the Yeast System

**Figure 11:**
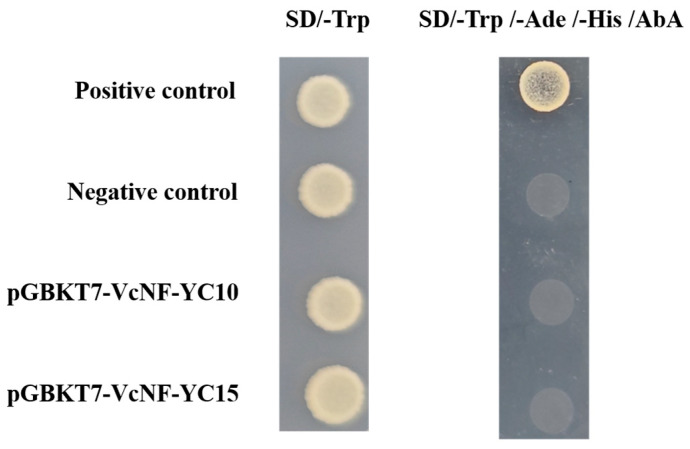
Transcription-activating activity of VcNF-YC10 and VcNF-YC15. SD/-Trp represents yeast culture medium deprived of tryptophan. SD/-Trp/-Ade/-His/AbA represents yeast culture medium deprived of tryptophan, adenine, and histidine, and supplemented with AbA (500 ng/L in medium). The positive control is the co-transformed pGBKT-53 + pGADT7-T, and the negative control is empty pGBKT7.

To evaluate the transcriptional activating activity of VcNF-YCs, VcNF-YC10 and VcNF-YC15, consistently responsive to ABA, salt, and cold treatments, underwent functional characterization via yeast two-hybrid assays. The full-length coding sequences of *VcNF-YC10* and *VcNF-YC15* were cloned into the pGBKT7 bait vector and transformed into yeast cells. As shown in Figure 11, neither negative control (empty pGBKT7) nor pGBKT7-VcNF-YA10/YA15 transformants grew under nutrition-deprived conditions (-Trp/-Ade/-His medium supplemented with AbA (Aureobasidin A)), whereas the positive control (pGBKT7-53 + pGADT7-T) showed robust growth. This indicates that VcNF-YC10 and VcNF-YC15 lack transcription-activating activity in the yeast system.

### 2.12. VcNF-YC10 and VcNF-YC15 Exhibit Nucleus Localization

**Figure 12:**
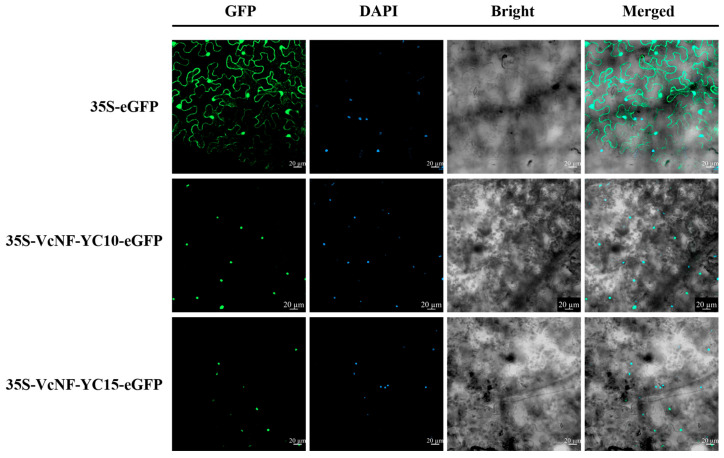
Subcellular localization of VcNF-YC10 and VcNF-YC15. GFP indicates the location of the green fluorescence, DAPI indicates the location of the nucleus, Bright indicates the bright field, and Merged indicates merged signals. Scale bar = 20 μm.

To determine subcellular localization, the coding sequences of *VcNF-YC10* and *VcNF-YC15* were fused to the pFGC-eGFP vector. Transient expression assays were conducted by infiltrating tobacco epidermal cells with *Agrobacterium tumefaciens* strains harboring either the empty pFGC-eGFP vector (negative control) or the recombinant constructs pFGC-eGFP-VcNF-YC10 and pFGC-eGFP-VcNF-YC15. Confocal microscopy observation revealed distinct localization patterns: eGFP fluorescence in control cells was ubiquitously distributed throughout the cytoplasm and nucleus. In contrast, eGFP-tagged VcNF-YC10 and VcNF-YC15 fusion proteins exhibited exclusive nuclear fluorescence (Figure 12). These results indicate that VcNF-YC10 and VcNF-YC15 are both localized in the nucleus.

## 3. Discussion

NF-Y TFs are evolutionarily conserved heterotrimeric complexes in eukaryotes, comprising three subunits: NF-YA, NF-YB, and NF-YC [39]. In plants, these subunits assemble to form a functional complex that binds specifically to CCAAT cis-elements in the promoters of target genes [40]. Unlike their animal counterparts, plant NF-Ys have undergone significant diversification, with expanded gene families, reflecting their adaptation to plant-specific regulatory demands [39]. The NF-YC subunit, as an integral component of the NF-Y complex, plays a critical role in modulating diverse biological processes, ranging from developmental programs to stress responses. For instance, Miao et al. identified *Cdt-NF-YC1* in bermudagrass, whose expression in leaves was induced by dehydration, salinity, ABA, and H_2_O_2_ treatments [41]. Overexpression of this gene in rice increased tolerance to drought and salt stress; similarly, *OsNF-YC5* was induced in rice by salt and ABA treatments [42]. The rice plants in which this gene was knocked out with the CRISPR-Cas9 method showed significantly enhanced salinity tolerance and ABA hypersensitivity. With the development of bioinformatics, NF-YC TFs have been identified in many plant species, including tobacco, wheat, and soybean [28,29,30]. Genome-wide characterization of *NF-YC* genes is essential for understanding stress adaptation in blueberry—an economically vital perennial crop with nutritional and ecological significance.

In this study, thirty-one *NF-YC* genes (designated *VcNF-YC1–VcNF-YC31*) were identified in the blueberry (*V. corymbosum*) genome. The number of *NF-YC* genes in blueberry exceeded the number in several economically important crops, including tobacco (16) [29], wheat (14) [28], soybean (15) [30], and grape (8) [35]. This intermediate expansion suggested adaptive diversification in woody perennials. All the VcNF-YCs harbored one NF-YB interaction and two NF-YC interaction conserved domains, while VcNF-YC13, -20, and -8 had incomplete NF-YC interaction domains. This indicates distinct functional roles of these three members. A comprehensive analysis of the physicochemical properties of the VcNF-YC family yielded crucial insights into their potential functional roles and structural stability. The general consistency in molecular weight (MW) and amino acid number across most members underscored the evolutionary conservation of the core histone-fold domain, which was indispensable for heterotrimeric complex formation with NF-YA and NF-YB. Exceptions such as VcNF-YC8 (7.5 kDa) and VcNF-YC13 (12.4 kDa) suggested that they may represent minimal functional units. The predicted isoelectric points (pIs) for the majority of VcNF-YCs were basic (pI > 7), indicating that they are DNA-binding proteins. The 31 VcNF-YCs were classified into five clades according to their phylogenetic relationships. VcNF-YCs shared closer phylogenetic relationships with AcNF-YCs than with AtNF-YCs, potentially resulting from the closer evolutionary relationships between the two woody plants; furthermore, members within the same phylogenetic clade exhibited conserved exon–intron architectures and motif compositions. The structural conservation observed among clade members may imply conserved functional roles in DNA binding or protein dimerization. This aligned with findings in *Arabidopsis*, where NF-YC paralogs with identical motif topologies exhibited redundant roles in flowering regulation [43]. In Clade 2, VcNF-YC23 and VcNF-YC21 shared closer phylogenetic relationships with AtNF-YC10 (AT1G07980). In *Arabidopsis*, AtNF-YC10 has been characterized as a key substrate for SUMOylation [44]. Specifically, the SUMO ligase SIZ1, mediating the SUMOylation of AtNF-YC10, was essential for the heat stress response. In this case, it can be hypothesized that VcNF-YC23 and VcNF-YC21 may play key roles under heat or other abiotic stresses in blueberries. The results of the intraspecies collinearity analysis showed that most (39/48) of the gene pairs underwent purification selection (Ka/Ks < 1.0 or both Ka and Ks are 0), while a small proportion (9/48) of the gene pairs underwent positive selection (Ka/Ks > 1.0). The prevalence of purifying selection among *VcNF-YC*s underscored the strong evolutionary constraints on this gene family [45]. The results of the intraspecies collinearity analysis revealed distinct evolutionary pressures on the duplicated *VcNF-YC* genes. The vast majority of gene pairs (39/48) exhibited a Ka/Ks ratio significantly less than 1, indicating that they have evolved under purifying selection. This strong evolutionary constraint underscores the fundamental importance of NF-YC proteins in core biological processes, such as transcription complex formation. In contrast, a small subset of gene pairs (9/48) showed evidence of positive selection (Ka/Ks > 1), suggesting that following duplication, these genes might have accumulated advantageous amino acid changes. This positive selection was often a hallmark of functional diversification, potentially in response to environmental pressures or developmental innovations specific to blueberry. The results of interspecies collinearity analysis showed that there were more collinear gene pairs between *VcNF-YC*s and *AcNF-YC*s and between *VcNF-YC*s and *PtNF-YC*s than between *VcNF-YC*s and *AcNF-YC*s. This could also be due to the closer evolutionary relationships between woody plants. Among *VcNF-YC*s, *VcNF-YC15*, *-4*, *-10*, *-11*, *-12*, and *-20* occurred with the highest frequencies in the collinear gene pairs. *VcNF-YC* genes with numerous collinear pairs across different plant species were likely evolutionarily conserved due to strong purifying selection. These genes may be responsible for encoding proteins critical for fundamental processes that are indispensable across plant lineages [46]. The *cis*-acting elements in the promoters of *VcNF-YC*s were mainly associated with phytohormone signaling, metabolism, and stress response, indicating the multifunctional roles of *VcNF-YC*s.

Tissue-specific expression profiling revealed preferential transcription of most of the *VcNF-YC*s in leaves compared to roots and stems, consistent with leaves serving as primary stress-sensing organs coordinating environmental responses [47]. Leaf-predominant expression enables rapid modulation of pigment metabolism and antioxidant systems in response to abiotic stresses [48]. For instance, Yang et al. identified the *LpPLDδ3* gene in *Lolium perenne*, which exhibited the highest expression level in leaves [49]. The perennial ryegrass protoplasts overexpressing this gene showed increased heat stress tolerance. Based on the enrichment of stress-related *cis*-elements in the promoters of *VcNF-YC*s, their transcriptional responses to ABA, NaCl, and cold treatments were quantified by qRT-PCR. Most of the *VcNF-YC*s were responsive to these treatments and exhibited rapid upregulation upon ABA and salt treatment. Half of the *VcNF-YC*s exhibited rapid upregulation in response to cold treatment. The fluctuating expression patterns of *VcNF-YC*s suggested their roles in the immediate response followed by precise attenuation, probably mediated by feedback mechanisms to fine-tune the downstream transcriptional output. These expression responses implied the potentially important functional roles of these genes in the abiotic stress tolerance of blueberry. In alfalfa (*Medicago sativa* L.), *MsNF-YC2* was shown to be induced by multiple abiotic stresses, including drought, salt, and alkali stress. Overexpression of this gene in alfalfa enhanced the alkaline stress tolerance of the transgenic plants by regulating the expression of phytohormone signal transduction and photosynthesis-related genes [50]. In soybean, the expression of *GmNF-YC14* was upregulated by drought, salt, and ABA treatments. CRISPR/Cas9-generated *GmNF-YC14* knockout mutants exhibited more sensitivity to drought compared to WT plants, while overexpression of this gene improved the drought tolerance of the transgenic plants [25]. Transcriptional activating activity was a basic property of TFs. The results of the yeast two-hybrid assay indicated that both VcNF-YC10 and VcNF-YC15 lacked transcriptional activating activity. This is consistent with prior research findings [13] and established critical baseline data for future protein interaction studies. The results suggest that they may act as obligate partners within the canonical NF-Y trimer, relying on NF-YA or other cofactors to provide the activation domain for gene expression; additionally, they may serve as repressive subunits that modulate the activity of other activating NF-Y trimers by forming non-productive complexes. Furthermore, the result of subcellular localization analysis indicated that both VcNF-YC10 and VcNF-YC15 were localized in the nucleus. This corroborates their roles as NF-Y complex subunits and core transcriptional regulators.

The findings of this study illuminate promising gene resources, *VcNF-YC* genes, for improving the stress tolerance of blueberry. Beyond genome-wide identification, this study provides a foundation for targeted functional characterization of *VcNF-YC* genes. A logical next step would be to generate transgenic blueberry plants or faster-growing model systems (e.g., tomato, *Arabidopsis*) using overexpression and CRISPR-Cas9-mediated gene editing. Targeting stress-responsive *VcNF-YC* genes through marker-assisted breeding or genetic engineering could enhance tolerance to abiotic stresses; simultaneously, leveraging their roles in fruit development and phytohormone signaling may optimize traits such as berry size, pigmentation, and postharvest longevity. By bridging genomic insights with agronomic needs, this work positions *VcNF-YC*s as pivotal candidates for sustaining and expanding blueberry cultivation. Future efforts should prioritize field trials of elite cultivars engineered at *VcNF-YC* loci, ensuring these discoveries translate into tangible benefits for food security.

## 4. Materials and Methods

### 4.1. Identification and Physicochemical Characterization of NF-YC Gene Family in Blueberry

Genomic resources for blueberry (*Vaccinium corymbosum*), including genome assembly, coding sequences (CDS), and protein annotations, were retrieved from the Vaccinium Genome Database (https://www.vaccinium.org/(accessed on 3 February 2025)). Two methods were adopted for the identification of *VcNF-YC* genes in the blueberry genome. First, the amino acid sequences of 14 *AtNF-YC* genes (AT1G07980.1, AT1G08970.1, AT1G54830.1, AT1G56170.1, AT3G12480.1, AT3G48590.1, AT5G19490.1, AT5G27910.1, AT5G38140.1, AT5G43250.1, AT5G50470.1, AT5G50480.1, AT5G50490.1, and AT5G63470.1) in *Arabidopsis thaliana* downloaded from the PlantTFDB v5.0 database (https://planttfdb.gao-lab.org/(accessed on 6 February 2025)) were used as query sequences to search for NF-YC members in *Vaccinium corymbosum* through BlastP [51] with an E-value cutoff of 1 × 10^-5^, a minimum query coverage of 50%, and a minimum identity of 40%. Second, after retrieving the HMM file of NF-YC (PF00808) from the Pfam database (http://pfam.xfam.org/ (accessed on 10 February 2025)), the HMMER v3.1 tool was used to identify putative VcNF-YC family members in the *V. corymbosum* database. The threshold for the HMMER E-value was 0.01. The physicochemical properties of VcNF-YC proteins, including molecular weight, isoelectric point (pI), aromaticity, instability index, aliphatic index, and GRAVY (Grand Average of Hydropathicity), were analyzed using ExPASy (http://www.expasy.org/(accessed on 20 February 2025)).

### 4.2. Multiple Sequence Alignment and Phylogenetic Analysis

Multiple sequence alignment was performed using TBtools (v2.136) [52]. A phylogenetic tree (1000 bootstrap replications) was constructed with NF-YC subfamily members in *V. corymbosum*, *Actinidia chinensis*, and *A. thaliana* with the Maximum Likelihood (ML) method using TBtools (v2.136) software.

### 4.3. Gene Structure and Conserved Motif Analysis

The exon–intron architectures of *VcNF-YC*s were mapped using the Gene Structure Display Server 2.0 (Gene Structure Display Server: http://gsds.cbi.pku.edu.cn/(accessed on 22 February 2025)) [53]. Conserved protein motifs in VcNF-YC sequences were identified using MEME Suite v5.0 (http://meme-suite.org/(accessed on 23 February 2025)) [54], with default parameters.

### 4.4. Intraspecies and Interspecies Collinearity Analysis and Ka/Ks Calculation

The Gff3 (General Feature Format 3) files of *A. thaiana* TAIR10, *A. chinensis* PS1, and *Populus trichocarpa* v4.1 were retrieved from Ensembl Plants [55] (http://plants.ensembl.org/index.html, accessed on 1 March 2025). The Gff3 files of *V. corymbosum* were retrieved from the Vaccinium Genome Database (https://www.vaccinium.org/ (accessed on 2 March 2025)). TBtools software was used to perform collinearity analysis and calculate Ka and Ks values.

### 4.5. Cis-Acting Element Analysis

The 2000 bp (base pair) promoter region upstream of each *VcNF-YC* gene was analyzed for *cis*-acting elements. The online tool New PLACE (https://www.dna.affrc.go.jp/PLACE/?action=newplace, accessed on 17 June 2025) was used for the identification of *cis*-acting elements. The results were visualized using TBtools.

### 4.6. Plant Materials and Treatments

Blueberry (*V. corymbosum* cv. ‘Duke’) seedlings used in this study were preserved by the School of Agriculture, Liaodong University (Dandong, China). They were cultivated in a greenhouse maintained at 25 °C with a 16/8 h light/dark photoperiod. After 60 days of hydroponic cultivation in MS medium, healthy blueberry seedlings at the vegetative growth stage (with approximately 6–8 fully expanded leaves and a well-developed root system) were selected for tissue-specific expression profiling. A fully independent experimental design was employed: for each stress treatment and each timepoint (3 h, 6 h, 12 h, and 24 h), a new set of plants was used. A single, common control group of untreated plants (0 h) was used for comparison with all stress treatments. For ABA or salt stress treatments, blueberry seedlings were subjected to ABA (100 µM) [56] or salt stress (200 mM NaCl) [57] treatments in hydroponic culture. The 3 h, 6 h, 12 h, and 24 h timepoints post-ABA/salt treatment were selected to capture the dynamic transcriptional profiles of the stress response [38]. For cold treatment, blueberry seedlings were pre-cultured in tissue culture containers for 30 days prior to cold treatment (4 °C for 3 h).

Following ABA, salt, and cold stress treatments, blueberry leaves were immediately sampled for further analysis. After removal of the attached culture medium, these samples were immediately frozen in liquid nitrogen and stored at −80 °C. Three biological replicates were set up during these processes.

### 4.7. RNA Extraction and Expression Analysis

RNA extraction was performed using an RNA extraction kit (Bioteke, Beijing, China). After electrophoresis examination, the RNA was reverse-transcribed into single-stranded cDNA using a reverse transcription kit (PrimeScript^TM^ RT reagent Kit, Takara Bio, Kusatsu, Japan). The obtained cDNA was diluted 10-fold to serve as the template for qRT-PCR analysis. qRT-PCR primers were designed using Primer 5.0 (Premier Biosoft, Palo Alto, CA, USA) based on the downloaded full-length cDNA sequences of *VcNF-YC*s. *GAPDH* was selected as the internal reference gene [58,59]. Three biological and three technical replicates were used. qRT-PCR was performed according to the method described by THUNDERBIRD Next SYBR qPCR Mix (TOYOBO, Osaka, Japan). The 20 µL reaction mixture was subjected to the following thermal cycling protocol: initial denaturation at 95 °C for 5 min, followed by 45 cycles of denaturation at 95 °C for 15 s and annealing/extension at 60 °C for 5 min. The reaction was carried out on an Applied Biosystems 7500 Fast Real-Time PCR System (Waltham, MA, USA). All reactions were repeated three times, and the relative gene expression levels were calculated with the 2^−ΔΔCt^ method [60]. Normalized count values were log_2_-transformed to stabilize variance and better meet the assumptions of normality underlying the statistical models used. Differences in significance were analyzed with Duncan’s multiple range test method (*p* < 0.05). Given the high sequence similarity among certain genes, a single primer pair was designed to simultaneously quantify a specific gene combination. The combinations included *VcNF-YC1/11/14*, *VcNF-YC5/9*, *VcNF-YC6/7/18*, and *VcNF-YC8/29*. The heatmaps were constructed using GraphPad Prism 9.0.0.

### 4.8. Transcriptional Activating Activity Analysis

VcNF-YC10 and VcNF-YC15 were selected as representative members to assess the transcriptional activating activity of VcNF-YCs. They were introduced into the pGBKT7 yeast expression vector. The recombinant vectors pGBKT7-VcNF-YC10 and pGBKT7-VcNF-YC15, as well as pGBKT-53/pGADT7-T (positive control) and pGBKT7 (negative control), were transformed into yeast competent cells (Y2H strain). After confirmation of successful transformation (normal growth on SD/-Trp medium), the yeast fluid was cultured on the nutrition-deprived (SD/-Trp/-Ade/-His) yeast culture medium for 3–5 d at 30 °C. The inclusion of X-α-Gal (40 mg/L in culture medium) and AbA (Aureobasidin A, 500 ng/L in culture medium) in the yeast culture medium provided more detectable evidence for the activation of the yeast GAL4 system.

### 4.9. Subcellular Localization Analysis

The full-length coding sequences of *VcNF-YC10* and *VcNF-YC15* without termination codons were amplified and introduced into the N-terminal of GFP driven by a CaMV35S promoter in the pFGC-eGFP vector. The two fusion vectors (35S-VcNF-YC10-eGFP and 35S-VcNF-YC15-eGFP) and negative control (35S-eGFP) were transformed into tobacco (*Nicotiana benthamiana*) epidermal cells. The GFP fluorescent images were photographed with a confocal microscope (ZEISS LSM 800, Shanghai, China) 48 h after transformation.

## 5. Conclusions

In this study, a total of 31 *NF-YC* genes (designated *VcNF-YC1–31*) were identified in the blueberry (*Vaccinium corymbosum*) genome. All VcNF-YCs possessed two NF-YC interaction domains and one NF-YB interaction domain. Phylogenetic analysis classified these *VcNF-YC* genes into five clades, with members in each clade exhibiting conserved exon–intron architectures and motif compositions. A total of 48 collinear gene pairs were identified between *VcNF-YC*s; in addition, 31, 40, and 50 collinear gene pairs were identified between blueberry and *Arabidopsis*, blueberry and poplar, and blueberry and Chinese kiwi fruit, respectively.

Promoter analysis further identified *cis*-acting elements enriched in phytohormone signaling, metabolism, and stress response. Expression profiling demonstrated leaf-predominant expression patterns and pronounced responsiveness to ABA, salt, and cold treatments. A majority of the *VcNF-YC*s exhibited rapid upregulation in response to ABA and salt treatments, while half of them exhibited upregulation in response to the cold treatment.

Notably, VcNF-YC10 and VcNF-YC15 lacked transcription-activating activity in the yeast GAL4 system and exhibited nucleus-localization. Collectively, these findings bridge the gap between model systems and woody perennials by revealing conserved yet distinct NF-YC-mediated stress response paradigms in blueberry, providing a framework for exploiting these mechanisms for enhancing abiotic resilience in fruit crops. This research also provides a scientific basis for developing resistant, high-yielding blueberry varieties that are adapted to diverse climatic conditions, thereby contributing to the stabilization of fruit production and long-term food security.

## Figures and Tables

**Table 1:** Physicochemical properties of the VcNF-YC transcription factors (TF).

Gene Name	Gene ID	Molecular Weight (Da)	Isoelectric Point	Amino Acid Number	Aromaticity	Instability Index	Aliphatic Index	GRAVY
*VcNF-YC1*	Vc_DUK_00003397-RA	28,992.86	6.2	258	0.0736	65.05	69.26	−0.57
*VcNF-YC2*	Vc_DUK_00003695-RA	27,852.48	5.95	248	0.0726	63.42	68.51	−0.61
*VcNF-YC3*	Vc_DUK_00005452-RA	24,215.23	4.83	218	0.0963	67.51	70.32	−0.5
*VcNF-YC4*	Vc_DUK_00006569-RA	25,930.56	4.77	233	0.0773	74.46	74.98	−0.39
*VcNF-YC5*	Vc_DUK_00007234-RA	29,442	5.29	261	0.0958	69.31	66.28	−0.66
*VcNF-YC6*	Vc_DUK_00015706-RA	30,370.61	4.81	273	0.044	44.1	66.89	−0.82
*VcNF-YC7*	Vc_DUK_00015861-RA	33,785.54	4.88	306	0.049	43.47	69.25	−0.73
*VcNF-YC8*	Vc_DUK_00019836-RA	7557.92	9.16	65	0.0615	49.66	108	−0.14
*VcNF-YC9*	Vc_DUK_00033502-RA	27,828.24	5.47	248	0.0887	68.27	68.19	−0.65
*VcNF-YC10*	Vc_DUK_00034072-RA	29,133.6	5.02	261	0.0881	68.25	85.17	−0.2
*VcNF-YC11*	Vc_DUK_00037277-RA	28,992.86	6.2	258	0.0736	65.05	69.26	−0.57
*VcNF-YC12*	Vc_DUK_00038716-RA	23,229.1	4.83	209	0.0861	70.02	71.48	−0.6
*VcNF-YC13*	Vc_DUK_00042431-RA	12,468.37	9.08	109	0.0642	58.72	83.21	−0.51
*VcNF-YC14*	Vc_DUK_00047914-RA	28,750.62	6.2	256	0.0742	64.74	69.8	−0.55
*VcNF-YC15*	Vc_DUK_00049583-RA	24,699.84	4.95	222	0.0991	65.41	74.77	−0.46
*VcNF-YC16*	Vc_DUK_00049963-RA	35,060.76	6.11	312	0.0833	64.47	67.56	−0.49
*VcNF-YC17*	Vc_DUK_00059976-RA	31,572	5.02	283	0.0459	42.72	64.88	−0.86
*VcNF-YC18*	Vc_DUK_00065880-RA	33,796.61	4.94	306	0.049	44.52	70.52	−0.71
*VcNF-YC19*	Vc_DUK_00070664-RA	29,147.63	5.02	261	0.0881	68.25	85.17	−0.2
*VcNF-YC20*	Vc_DUK_00074190-RA	10,697.01	3.97	99	0.1313	57.23	67.07	−0.15
*VcNF-YC21*	Vc_DUK_00078307-RA	25,912.97	4.97	231	0.039	58.41	61.99	−1.07
*VcNF-YC22*	Vc_DUK_00089151-RA	31,254.26	6.49	277	0.0866	73.62	69.13	−0.64
*VcNF-YC23*	Vc_DUK_00092634-RA	25,051.94	5.07	223	0.0359	61.04	59.42	−1.16
*VcNF-YC24*	Vc_DUK_00093707-RA	13,244.08	7.23	121	0.0496	56.21	83.72	−0.26
*VcNF-YC25*	Vc_DUK_00093708-RA	13,229.06	6.48	120	0.05	58.85	84.42	−0.29
*VcNF-YC26*	Vc_DUK_00095693-RA	29,133.6	5.02	261	0.0881	68.25	85.17	−0.2
*VcNF-YC27*	Vc_DUK_00101188-RA	15,209.75	6.07	134	0.0522	64.72	104.78	−0.15
*VcNF-YC28*	Vc_DUK_00101294-RA	35,098.75	5.97	312	0.0833	64.4	67.56	−0.51
*VcNF-YC29*	Vc_DUK_00101495-RA	14,655.99	7.89	129	0.0465	62.19	89.92	−0.43
*VcNF-YC30*	Vc_DUK_00102060-RA	41,228.12	5.55	367	0.0899	56.7	77.6	−0.28
*VcNF-YC31*	Vc_DUK_00103149-RA	31,121.6	5.59	281	0.0463	50	59.32	−0.88

Gene Name

Gene ID

Molecular Weight (Da)

Isoelectric Point

Amino Acid Number

Aromaticity

Instability Index

Aliphatic Index

GRAVY

## Data Availability

Data is contained within the article and Supplementary Material.

## Supplementary Materials

The supporting information can be downloaded at: https://www.mdpi.com/article/10.3390/ijms26178507/s1.
